# Tris{*N*-[bis­(dimethyl­amino)phosphino­yl]-2,2,2-trichloro­acetamido}(triphenyl­phosphine oxide)holmium(III)

**DOI:** 10.1107/S160053681001665X

**Published:** 2010-05-12

**Authors:** Oleksiy V. Amirkhanov, Ivan O. Marchenko, Olesia V. Moroz, Tetyana Yu. Sliva, Igor O. Fritsky

**Affiliations:** a Kyiv National Taras Shevchenko University, Department of Chemistry, Volodymyrska str. 64, 01601 Kyiv, Ukraine

## Abstract

In the title compound, [Ho(C_6_H_12_Cl_3_N_3_O_2_P)_3_(C_18_H_15_OP)], the Ho^III^ ion is surrounded by six O atoms from the three bidentate *N*-[bis­(dimethyl­amino)phosphino­yl]-2,2,2-trichloro­acetamido ligands (*L* ^−^) and by one O atom from the triphenyl­phosphine oxide ligand, with the formation of a distorted monocapped octa­hedron. In one ligand *L* ^−^, the trichloro­methyl group is rotationally disordered between two orientations in a 1:1 ratio, while two dimethyl­amino groups in another ligand *L* ^−^ are disordered between two conformations, each with the same 1:1 ratio.

## Related literature

For the synthesis and structural investigation of *N*-[bis­(dimethyl­amino)phosphino­yl]-2,2,2-trichloro­acetamide, see: Amir­khanov *et al.* (2010 ▶). For *Ln* ^III^  (*Ln* = lanthanide) complexes with a triphenyl­phosphine oxide, see: Zhong *et al.* (2006 ▶); Cao *et al.* (2005 ▶). For the method of calculation of the coordination polyhedra of *Ln* ions, see: Kouba & Wreford (1976 ▶). For details of the potential application of lanthanide complexes, see: Bünzli & Piguet (2005 ▶). For *Ln* ^III^ complexes with CAPh-type (carbacylamidophosphate) ligands, see: Borzechowska *et al.* (2002 ▶); Trush *et al.* (2001 ▶); Znovjyak *et al.* (2009 ▶).
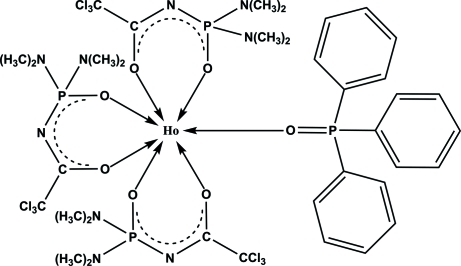


## Experimental

### 

#### Crystal data

[Ho(C_6_H_12_Cl_3_N_3_O_2_P)_3_(C_18_H_15_OP)]
*M* *_r_* = 1329.72
Monoclinic, 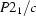

*a* = 12.1338 (4) Å
*b* = 23.2403 (9) Å
*c* = 23.6071 (8) Å
β = 120.462 (2)°
*V* = 5738.1 (4) Å^3^
*Z* = 4
Mo *K*α radiation
μ = 1.96 mm^−1^
*T* = 293 K
0.10 × 0.07 × 0.03 mm

#### Data collection

Nonius KappaCCD diffractometer
Absorption correction: multi-scan (*SADABS*; Sheldrick, 2003 ▶) *T*_min_ = 0.848, *T*_max_ = 0.943
24428 measured reflections
10386 independent reflections
7799 reflections with *I* > 2σ(*I*)
*R*_int_ = 0.021

#### Refinement

*R*[*F* ^2^ > 2σ(*F* ^2^)] = 0.041
*wR*(*F* ^2^) = 0.120
*S* = 1.09
10386 reflections
668 parameters
120 restraints
H-atom parameters constrained
Δρ_max_ = 0.81 e Å^−3^
Δρ_min_ = −0.76 e Å^−3^

### 

Data collection: *COLLECT* (Nonius, 1999 ▶); cell refinement: *DENZO*/*SCALEPACK* (Otwinowski & Minor, 1997 ▶); data reduction: *DENZO*/*SCALEPACK*; program(s) used to solve structure: *SHELXS97* (Sheldrick, 2008 ▶); program(s) used to refine structure: *SHELXL97* (Sheldrick, 2008 ▶); molecular graphics: *ORTEP-3 for Windows* (Farrugia, 1997 ▶); software used to prepare material for publication: *WinGX* (Farrugia, 1999 ▶).

## Supplementary Material

Crystal structure: contains datablocks I, global. DOI: 10.1107/S160053681001665X/cv2713sup1.cif

Structure factors: contains datablocks I. DOI: 10.1107/S160053681001665X/cv2713Isup2.hkl

Additional supplementary materials:  crystallographic information; 3D view; checkCIF report

## Figures and Tables

**Fig. 1.**
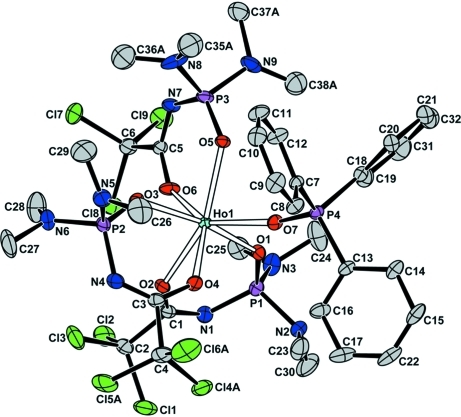
Molecular structure of 1 with atom numbering scheme and 20% probability displacement ellipsoids. For each disordered atom, only one position (labelled with letter A) is shown. H atoms were omitted for clarity.

## supplementary crystallographic information

### Comment

Synthesis of luminescent lanthanide complexes has been attracted a considerable interest because of their potential application, such as fluorescent labeling reagents, imaging agents, and emitter materials in organic light-emitting diodes (Bünzli & Piguet, 2005). As a part of our study of Ln(III) coordination compounds based on carbacylamidophosphates (CAPh), which comprise C(O)NHP(O) structural fragment, we synthesized and structurally characterized compound [Ho^III^(*L*^-^)_3_TPPO] (1) {H*L* is *N*-[bis(dimethylamino)phosphinoyl]-2,2,2-trichloroacetamide, TPPO is triphenylphosphine oxide}.

The molecular structure of 1 is shown in Fig. 1. There are no short contacts between neighboring molecules in the crystal packing. The coordination environment of Ho^III^ ion can be described as a distorted monocapped octahedron polyhedron (6 + 1) (Kouba & Wreford, 1976). The HoO_7_ center is made by one oxygen atom from TPPO molecule and six oxygen atoms from phosphoryl and carbonyl groups from three ligands (*L*^-^) which are coordinated in bidentate chelate mode forming with central ion six-membered chelate rings.

The Ho–O(P) and Ho–O(C) distances from *L*^-^ fall in the range 2.241 (3) – 2.282 (3) Å and 2.322 (4) – 2.330 (4) Å, respectively. The bond lengths Ho–O(P) are shorter than Ho–O(C) which is a result of higher affinity of phosphoryl group to lanthanides. The Ho–O(TPPO) bond distance is 2.258 (4) Å which is similar to values observed for complexes with TPPO ligand (Zhong *et al.*, 2006; Cao *et al.*, 2005). The amide nitrogen atom of *L*^-^ is deprotonated that leads to decrease of C–N, N–P and increase of P–O, C–O bond length values in comparison with the same values for neutral ligand (Amirkhanov *et al.*, 2010). Such changes of the bond lengths may be related to the occurrence of the *π*-coupling in C(O)NP(O) fragment (Znovjyak *et al.*, 2009).

The bite angles around the central atom lie in the range 75.4 (1) – 76.3 (1)° which are typical for lanthanide complexes with O- donor ligands (Trush *et al.*, 2001; Borzechowska *et al.*, 2002). The phosphorus atoms of the complex 1 have distorted tetrahedral configuration. The O–P–N chelate angle has value 116.2 (2)°. The sum of the contiguous angles of O–C–N chelate angle is 360°, which is expected for the sp^2^-hybridization of C atom.

### Experimental

The synthesis of H*L* was carried out according to previously published procedure (Amirkhanov *et al.*, 2010).

A solution of H*L* (0.89 g., 3 mmol) in isopropanol (10 ml) was mixed with isopropanol solution of sodium (0.07 g., 3 mmol). After that acetone solution of Ho(NO_3_)_3_^.^6H_2_O (5 ml) (0.46 g., 1 mmol) was added. After 20 min the precipitate of NaNO_3_ was filtered off and filtrate was added to 10 ml of isopropanol solution of TPPO (0.28 g., 1 mmol). The resulting clear solution was left at ambient temperature for crystallization in air. The yellow crystals were collected by filtration after 5 days, washed thoroughly with cool isopropanol and finally dried on filter. A well-formed crystal was used for the single X-ray structural analysis. Yield: 1.06 g (80%). IR (KBr pellet, cm^-1^): 1608 (ν(CO)), 1125 (ν(PO)), 1105 (ν(PO)), 993 (ν(PN_amine_)), 870 (ν(PN_amide_)), 668 (ν(CCl)).

### Refinement

The H atoms were geometrically positioned (C—H 0.93–0.98Å), and refined as riding, with U_iso_(H) = 1.2-1.5 U_eq_ of the parent atom. Atoms Cl4, Cl5, Cl6, C35, C36, C37 and C38 were treated as disordered between two positions each   with occupancy factors fixed to 0.5. In the refinement, several constrains were applied: SIMU and ISOR for CL4A CL4B CL6A CL6B CL5A CL5B atoms; ISOR for disordered C atoms of one NMe_2_ group; EADP instruction - for O6 C5 atoms. Also several ISOR and DFIX instructions were added.

### Crystal data

**Table:**

[Ho(C_6_H_12_Cl_3_N_3_O_2_P)_3_(C_18_H_15_OP)]	*F*(000) = 2664
*M**_r_* = 1329.72	*D*_x_ = 1.539 Mg m^−^^3^
Monoclinic, *P*2_1_/*c*	Mo *K*α radiation, λ = 0.71073 Å
Hall symbol: -P 2ybc	Cell parameters from 24428 reflections
*a* = 12.1338 (4) Å	θ = 1.8–26.0°
*b* = 23.2403 (9) Å	µ = 1.96 mm^−^^1^
*c* = 23.6071 (8) Å	*T* = 293 K
β = 120.462 (2)°	Block, colorless
*V* = 5738.1 (4) Å^3^	0.1 × 0.07 × 0.03 mm
*Z* = 4	

### Data collection

**Table:**

Nonius KappaCCD diffractometer	10386 independent reflections
Radiation source: fine-focus sealed tube	7799 reflections with *I* > 2σ(*I*)
horizontally mounted graphite crystal	*R*_int_ = 0.021
Detector resolution: 9 pixels mm^-1^	θ_max_ = 26.0°, θ_min_ = 1.8°
φ scans and ω scans with κ offset	*h* = −14→14
Absorption correction: multi-scan (*SADABS*; Sheldrick, 2003)	*k* = −28→20
*T*_min_ = 0.848, *T*_max_ = 0.943	*l* = −27→24
24428 measured reflections	

### Refinement

**Table:**

Refinement on *F*^2^	Primary atom site location: structure-invariant direct methods
Least-squares matrix: full	Secondary atom site location: difference Fourier map
*R*[*F*^2^ > 2σ(*F*^2^)] = 0.041	Hydrogen site location: inferred from neighbouring sites
*wR*(*F*^2^) = 0.120	H-atom parameters constrained
*S* = 1.09	*w* = 1/[σ^2^(*F*_o_^2^) + (0.0588*P*)^2^ + 5.5377*P*] where *P* = (*F*_o_^2^ + 2*F*_c_^2^)/3
10386 reflections	(Δ/σ)_max_ = 0.001
668 parameters	Δρ_max_ = 0.81 e Å^−^^3^
120 restraints	Δρ_min_ = −0.76 e Å^−^^3^

### Special details

**Table:**

Geometry. All s.u.'s (except the s.u. in the dihedral angle between two l.s. planes) are estimated using the full covariance matrix. The cell s.u.'s are taken into account individually in the estimation of s.u.'s in distances, angles and torsion angles; correlations between s.u.'s in cell parameters are only used when they are defined by crystal symmetry. An approximate (isotropic) treatment of cell s.u.'s is used for estimating s.u.'s involving l.s. planes.
Refinement. Refinement of *F*^2^ against ALL reflections. The weighted *R*-factor wR and goodness of fit *S* are based on *F*^2^, conventional *R*-factors *R* are based on *F*, with *F* set to zero for negative *F*^2^. The threshold expression of *F*^2^ > 2σ(*F*^2^) is used only for calculating *R*-factors(gt) etc. and is not relevant to the choice of reflections for refinement. *R*-factors based on *F*^2^ are statistically about twice as large as those based on *F*, and *R*- factors based on ALL data will be even larger.

### Fractional atomic coordinates and isotropic or equivalent isotropic displacement parameters (Å^2^)

**Table:**

	*x*	*y*	*z*	*U*_iso_*/*U*_eq_	Occ. (<1)
Ho1	−0.72329 (2)	−0.161007 (10)	0.243739 (10)	0.04697 (10)	
Cl1	−0.9056 (3)	−0.34718 (11)	0.05525 (12)	0.1383 (10)	
Cl2	−0.7085 (3)	−0.39845 (10)	0.17412 (13)	0.1391 (9)	
Cl3	−0.6463 (3)	−0.30681 (14)	0.11674 (17)	0.1613 (12)	
Cl4A	−0.9802 (6)	−0.1737 (3)	0.0109 (5)	0.124 (2)	0.50
Cl5A	−0.7709 (8)	−0.1689 (3)	−0.0115 (4)	0.174 (2)	0.50
Cl6A	−0.8820 (11)	−0.0633 (3)	0.0032 (7)	0.200 (3)	0.50
Cl4B	−0.9374 (10)	−0.0728 (3)	0.0048 (7)	0.201 (3)	0.50
Cl5B	−0.9406 (6)	−0.1955 (3)	0.0120 (6)	0.129 (2)	0.50
Cl6B	−0.7802 (8)	−0.1343 (4)	−0.0245 (4)	0.171 (2)	0.50
Cl7	−0.2850 (2)	−0.28801 (12)	0.43120 (13)	0.1385 (9)	
Cl8	−0.5130 (3)	−0.34413 (9)	0.33699 (13)	0.1338 (10)	
Cl9	−0.4751 (3)	−0.31821 (11)	0.46425 (12)	0.1291 (8)	
P1	−0.94063 (15)	−0.26903 (7)	0.21883 (8)	0.0660 (4)	
P2	−0.51228 (16)	−0.12129 (8)	0.19290 (9)	0.0758 (5)	
P3	−0.52002 (18)	−0.12899 (8)	0.40950 (7)	0.0770 (5)	
P4	−0.93045 (14)	−0.03620 (6)	0.22533 (7)	0.0602 (4)	
O1	−0.8846 (4)	−0.21046 (16)	0.24089 (19)	0.0664 (10)	
O2	−0.7405 (4)	−0.24256 (17)	0.18238 (19)	0.0673 (10)	
O3	−0.5430 (4)	−0.1362 (2)	0.2448 (2)	0.0801 (12)	
O4	−0.7958 (4)	−0.1297 (2)	0.13714 (19)	0.0746 (11)	
O5	−0.6267 (4)	−0.11480 (16)	0.34270 (17)	0.0640 (10)	
O6	−0.5990 (4)	−0.23135 (17)	0.31940 (18)	0.0783 (10)	
O7	−0.8631 (4)	−0.08973 (16)	0.22698 (19)	0.0706 (11)	
N1	−0.8772 (5)	−0.3085 (2)	0.1869 (3)	0.0770 (14)	
N2	−1.0926 (5)	−0.2607 (3)	0.1671 (3)	0.0885 (16)	
N3	−0.9369 (6)	−0.3071 (3)	0.2769 (4)	0.103 (2)	
N4	−0.6283 (6)	−0.1282 (3)	0.1180 (3)	0.0846 (16)	
N5	−0.4640 (7)	−0.0553 (3)	0.1967 (4)	0.107 (2)	
N6	−0.3919 (7)	−0.1609 (3)	0.2046 (4)	0.117 (2)	
N7	−0.4672 (5)	−0.1943 (2)	0.4212 (2)	0.0832 (16)	
N8	−0.3974 (7)	−0.0871 (3)	0.4310 (4)	0.142 (3)	
N9	−0.5635 (8)	−0.1163 (4)	0.4628 (3)	0.130 (3)	
C1	−0.7961 (6)	−0.2894 (3)	0.1711 (3)	0.0641 (15)	
C2	−0.7642 (8)	−0.3329 (3)	0.1308 (4)	0.091 (2)	
C3	−0.7447 (7)	−0.1314 (3)	0.1030 (3)	0.0744 (17)	
C4	−0.8432 (4)	−0.13507 (15)	0.0277 (2)	0.116 (3)	
C5	−0.5139 (6)	−0.2329 (3)	0.3773 (3)	0.0783 (10)	
C6	−0.4505 (7)	−0.2931 (3)	0.4009 (3)	0.092 (2)	
C7	−0.8378 (5)	0.0255 (2)	0.2310 (3)	0.0642 (14)	
C8	−0.8698 (6)	0.0611 (3)	0.1787 (3)	0.0835 (19)	
H8	−0.9459	0.0552	0.1394	0.100*	
C9	−0.7895 (9)	0.1058 (3)	0.1839 (5)	0.108 (3)	
H9	−0.8124	0.1297	0.1480	0.129*	
C10	−0.6777 (9)	0.1151 (3)	0.2409 (5)	0.106 (3)	
H10	−0.6236	0.1449	0.2442	0.127*	
C11	−0.6463 (8)	0.0797 (4)	0.2935 (5)	0.114 (3)	
H11	−0.5711	0.0864	0.3331	0.137*	
C12	−0.7232 (7)	0.0349 (3)	0.2888 (4)	0.101 (2)	
H12	−0.6986	0.0106	0.3245	0.121*	
C13	−1.0815 (5)	−0.0322 (3)	0.1507 (3)	0.0691 (16)	
C14	−1.1820 (6)	−0.0009 (4)	0.1460 (4)	0.104 (3)	
H14	−1.1734	0.0190	0.1822	0.125*	
C15	−1.2971 (8)	0.0008 (5)	0.0862 (6)	0.138 (4)	
H15	−1.3647	0.0228	0.0821	0.166*	
C16	−1.0962 (8)	−0.0609 (3)	0.0970 (4)	0.100 (2)	
H16	−1.0282	−0.0816	0.0996	0.120*	
C17	−1.2128 (11)	−0.0594 (4)	0.0381 (4)	0.138 (4)	
H17	−1.2223	−0.0791	0.0016	0.165*	
C18	−0.9605 (6)	−0.0337 (3)	0.2918 (3)	0.0791 (18)	
C19	−0.9759 (8)	−0.0857 (4)	0.3150 (4)	0.112 (3)	
H19	−0.9681	−0.1201	0.2972	0.134*	
C20	−0.9716 (7)	0.0171 (4)	0.3191 (4)	0.112 (3)	
H20	−0.9614	0.0522	0.3033	0.134*	
C21	−0.9981 (9)	0.0159 (7)	0.3707 (6)	0.156 (5)	
H21	−1.0059	0.0496	0.3896	0.187*	
C22	−1.3108 (9)	−0.0297 (5)	0.0338 (6)	0.141 (4)	
H22	−1.3891	−0.0299	−0.0052	0.170*	
C23	−1.1234 (9)	−0.2171 (5)	0.1167 (4)	0.150 (4)	
H23A	−1.1074	−0.2323	0.0837	0.226*	
H23B	−1.0711	−0.1837	0.1363	0.226*	
H23C	−1.2119	−0.2067	0.0968	0.226*	
C24	−0.9846 (12)	−0.2795 (5)	0.3164 (6)	0.169 (5)	
H24A	−1.0106	−0.3086	0.3362	0.253*	
H24B	−1.0563	−0.2555	0.2887	0.253*	
H24C	−0.9179	−0.2566	0.3502	0.253*	
C25	−0.8443 (11)	−0.3483 (4)	0.3128 (5)	0.136 (4)	
H25A	−0.7796	−0.3318	0.3535	0.205*	
H25B	−0.8062	−0.3613	0.2880	0.205*	
H25C	−0.8831	−0.3803	0.3218	0.205*	
C26	−0.5602 (14)	−0.0100 (4)	0.1656 (7)	0.179 (5)	
H26A	−0.5833	0.0042	0.1964	0.268*	
H26B	−0.6346	−0.0254	0.1279	0.268*	
H26C	−0.5259	0.0209	0.1522	0.268*	
C27	−0.3484 (13)	−0.1568 (6)	0.1575 (7)	0.200 (7)	
H27A	−0.2573	−0.1519	0.1806	0.301*	
H27B	−0.3884	−0.1244	0.1292	0.301*	
H27C	−0.3708	−0.1913	0.1318	0.301*	
C28	−0.3516 (12)	−0.2104 (5)	0.2451 (7)	0.191 (6)	
H28A	−0.3652	−0.2439	0.2186	0.287*	
H28B	−0.4000	−0.2138	0.2669	0.287*	
H28C	−0.2624	−0.2071	0.2773	0.287*	
C29	−0.3499 (12)	−0.0355 (5)	0.2495 (6)	0.191 (6)	
H29A	−0.3103	−0.0091	0.2340	0.286*	
H29B	−0.2937	−0.0675	0.2706	0.286*	
H29C	−0.3670	−0.0164	0.2802	0.286*	
C30	−1.1715 (10)	−0.3112 (5)	0.1432 (7)	0.191 (6)	
H30A	−1.2589	−0.3010	0.1275	0.287*	
H30B	−1.1438	−0.3387	0.1782	0.287*	
H30C	−1.1643	−0.3278	0.1080	0.287*	
C31	−1.0041 (11)	−0.0862 (7)	0.3671 (6)	0.167 (5)	
H31	−1.0166	−0.1205	0.3834	0.200*	
C32	−1.0116 (15)	−0.0354 (8)	0.3911 (8)	0.175 (6)	
H32	−1.0279	−0.0359	0.4256	0.210*	
C35A	−0.401 (3)	−0.0269 (4)	0.4280 (16)	0.197 (12)	0.50
H35A	−0.3405	−0.0135	0.4159	0.296*	0.50
H35B	−0.3785	−0.0116	0.4702	0.296*	0.50
H35C	−0.4850	−0.0144	0.3958	0.296*	0.50
C36A	−0.2863 (18)	−0.1091 (11)	0.4349 (14)	0.184 (10)	0.50
H36A	−0.2894	−0.1029	0.3939	0.276*	0.50
H36B	−0.2803	−0.1496	0.4441	0.276*	0.50
H36C	−0.2129	−0.0899	0.4695	0.276*	0.50
C37A	−0.452 (2)	−0.1140 (10)	0.5350 (9)	0.145 (7)	0.50
H37A	−0.4657	−0.0825	0.5572	0.217*	0.50
H37B	−0.3730	−0.1088	0.5360	0.217*	0.50
H37C	−0.4497	−0.1494	0.5567	0.217*	0.50
C38A	−0.687 (2)	−0.1197 (14)	0.4493 (13)	0.161 (9)	0.50
H38A	−0.6958	−0.0978	0.4813	0.242*	0.50
H38B	−0.7084	−0.1591	0.4510	0.242*	0.50
H38C	−0.7433	−0.1044	0.4063	0.242*	0.50
C35B	−0.386 (2)	−0.0480 (10)	0.3891 (10)	0.151 (8)	0.50
H35D	−0.3569	−0.0116	0.4110	0.227*	0.50
H35E	−0.4675	−0.0432	0.3499	0.227*	0.50
H35F	−0.3254	−0.0625	0.3778	0.227*	0.50
C36B	−0.316 (2)	−0.0848 (11)	0.4992 (4)	0.177 (9)	0.50
H36D	−0.2953	−0.0454	0.5128	0.266*	0.50
H36E	−0.2389	−0.1057	0.5112	0.266*	0.50
H36F	−0.3578	−0.1017	0.5205	0.266*	0.50
C37B	−0.622 (3)	−0.1630 (10)	0.4744 (18)	0.209 (14)	0.50
H37D	−0.6364	−0.1538	0.5099	0.314*	0.50
H37E	−0.5677	−0.1962	0.4860	0.314*	0.50
H37F	−0.7025	−0.1711	0.4355	0.314*	0.50
C38B	−0.630 (2)	−0.0701 (7)	0.4695 (11)	0.141 (7)	0.50
H38D	−0.7192	−0.0732	0.4369	0.212*	0.50
H38E	−0.5969	−0.0346	0.4635	0.212*	0.50
H38F	−0.6200	−0.0709	0.5125	0.212*	0.50

### Atomic displacement parameters (Å^2^)

**Table:**

	*U*^11^	*U*^22^	*U*^33^	*U*^12^	*U*^13^	*U*^23^
Ho1	0.04653 (15)	0.04951 (15)	0.03866 (14)	−0.00049 (11)	0.01704 (11)	0.00195 (10)
Cl1	0.156 (2)	0.143 (2)	0.0812 (14)	−0.0087 (16)	0.0353 (15)	−0.0423 (13)
Cl2	0.161 (2)	0.0863 (14)	0.145 (2)	0.0377 (14)	0.0598 (18)	−0.0174 (14)
Cl3	0.184 (3)	0.162 (2)	0.215 (3)	−0.031 (2)	0.158 (3)	−0.072 (2)
Cl4A	0.085 (4)	0.188 (5)	0.0807 (18)	−0.032 (4)	0.028 (3)	−0.019 (4)
Cl5A	0.198 (3)	0.276 (6)	0.077 (3)	−0.072 (4)	0.090 (3)	−0.024 (4)
Cl6A	0.207 (7)	0.215 (4)	0.114 (2)	−0.006 (4)	0.034 (4)	0.097 (3)
Cl4B	0.209 (7)	0.219 (4)	0.113 (3)	0.003 (4)	0.036 (5)	0.091 (3)
Cl5B	0.091 (4)	0.188 (5)	0.0810 (18)	−0.032 (4)	0.024 (3)	−0.016 (4)
Cl6B	0.198 (3)	0.273 (6)	0.074 (3)	−0.077 (4)	0.092 (3)	−0.015 (4)
Cl7	0.0878 (14)	0.167 (2)	0.1322 (19)	0.0585 (15)	0.0351 (13)	0.0100 (17)
Cl8	0.160 (2)	0.0875 (14)	0.1074 (17)	0.0429 (14)	0.0341 (16)	−0.0088 (11)
Cl9	0.160 (2)	0.1119 (16)	0.1042 (16)	0.0313 (16)	0.0591 (16)	0.0469 (14)
P1	0.0615 (9)	0.0623 (9)	0.0765 (10)	−0.0070 (7)	0.0366 (8)	0.0062 (8)
P2	0.0708 (10)	0.0889 (12)	0.0823 (12)	−0.0165 (9)	0.0495 (10)	−0.0190 (9)
P3	0.0857 (12)	0.0771 (11)	0.0430 (8)	0.0138 (9)	0.0142 (8)	−0.0063 (8)
P4	0.0509 (8)	0.0558 (8)	0.0596 (9)	0.0070 (7)	0.0176 (7)	0.0044 (7)
O1	0.076 (3)	0.061 (2)	0.080 (3)	−0.013 (2)	0.051 (2)	−0.006 (2)
O2	0.081 (3)	0.067 (2)	0.064 (2)	−0.014 (2)	0.044 (2)	−0.016 (2)
O3	0.051 (2)	0.122 (4)	0.066 (3)	−0.015 (2)	0.029 (2)	−0.011 (2)
O4	0.069 (3)	0.102 (3)	0.049 (2)	0.007 (2)	0.028 (2)	0.021 (2)
O5	0.069 (2)	0.063 (2)	0.044 (2)	0.0124 (19)	0.0172 (18)	−0.0039 (17)
O6	0.088 (3)	0.061 (2)	0.0512 (18)	0.0130 (19)	0.0098 (17)	0.0016 (17)
O7	0.068 (2)	0.058 (2)	0.071 (3)	0.0159 (19)	0.024 (2)	0.0095 (19)
N1	0.080 (4)	0.054 (3)	0.098 (4)	−0.009 (3)	0.046 (3)	−0.008 (3)
N2	0.062 (3)	0.109 (4)	0.085 (4)	−0.008 (3)	0.030 (3)	−0.009 (3)
N3	0.106 (5)	0.092 (4)	0.131 (6)	0.010 (4)	0.076 (4)	0.045 (4)
N4	0.094 (4)	0.100 (4)	0.076 (4)	−0.029 (3)	0.054 (3)	−0.011 (3)
N5	0.101 (5)	0.099 (5)	0.132 (6)	−0.035 (4)	0.068 (5)	−0.036 (4)
N6	0.098 (5)	0.139 (7)	0.140 (7)	0.011 (4)	0.078 (5)	−0.016 (5)
N7	0.095 (4)	0.079 (4)	0.050 (3)	0.025 (3)	0.018 (3)	0.004 (3)
N8	0.100 (5)	0.115 (6)	0.105 (6)	−0.021 (5)	−0.026 (4)	−0.002 (5)
N9	0.152 (7)	0.163 (7)	0.051 (4)	0.062 (6)	0.035 (4)	0.000 (4)
C1	0.069 (4)	0.057 (3)	0.055 (3)	0.001 (3)	0.024 (3)	−0.007 (3)
C2	0.099 (5)	0.075 (4)	0.093 (5)	−0.001 (4)	0.045 (4)	−0.026 (4)
C3	0.086 (5)	0.083 (4)	0.055 (4)	−0.011 (4)	0.037 (3)	0.004 (3)
C4	0.119 (6)	0.171 (8)	0.051 (4)	−0.032 (6)	0.038 (4)	0.015 (5)
C5	0.088 (3)	0.061 (2)	0.0512 (18)	0.0130 (19)	0.0098 (17)	0.0016 (17)
C6	0.097 (5)	0.088 (5)	0.067 (4)	0.032 (4)	0.024 (4)	0.011 (4)
C7	0.056 (3)	0.056 (3)	0.069 (4)	0.003 (3)	0.023 (3)	−0.001 (3)
C8	0.070 (4)	0.084 (5)	0.082 (5)	−0.005 (4)	0.028 (4)	0.016 (4)
C9	0.110 (6)	0.088 (5)	0.124 (7)	−0.002 (5)	0.058 (6)	0.029 (5)
C10	0.104 (6)	0.074 (5)	0.145 (8)	−0.022 (5)	0.066 (6)	−0.009 (5)
C11	0.087 (5)	0.123 (7)	0.107 (7)	−0.044 (5)	0.031 (5)	−0.014 (6)
C12	0.078 (5)	0.111 (6)	0.080 (5)	−0.022 (4)	0.016 (4)	0.011 (4)
C13	0.053 (3)	0.056 (3)	0.073 (4)	−0.003 (3)	0.013 (3)	0.006 (3)
C14	0.058 (4)	0.122 (6)	0.105 (6)	0.012 (4)	0.021 (4)	0.021 (5)
C15	0.053 (5)	0.181 (11)	0.132 (9)	0.023 (6)	0.012 (5)	0.026 (8)
C16	0.093 (5)	0.084 (5)	0.078 (5)	0.010 (4)	0.009 (4)	0.004 (4)
C17	0.129 (8)	0.114 (7)	0.086 (6)	−0.024 (7)	−0.007 (6)	−0.007 (5)
C18	0.056 (4)	0.103 (5)	0.074 (4)	0.004 (3)	0.030 (3)	0.000 (4)
C19	0.105 (6)	0.136 (8)	0.109 (6)	0.000 (5)	0.065 (5)	0.021 (6)
C20	0.090 (5)	0.142 (8)	0.114 (7)	−0.006 (5)	0.059 (5)	−0.034 (6)
C21	0.098 (7)	0.220 (14)	0.147 (10)	−0.014 (8)	0.060 (7)	−0.083 (10)
C22	0.064 (6)	0.153 (10)	0.126 (9)	−0.014 (6)	−0.011 (6)	0.022 (7)
C23	0.101 (6)	0.247 (13)	0.101 (7)	0.055 (8)	0.050 (5)	0.065 (8)
C24	0.236 (13)	0.159 (10)	0.206 (12)	0.059 (9)	0.182 (11)	0.076 (9)
C25	0.162 (10)	0.122 (8)	0.126 (8)	0.027 (7)	0.075 (8)	0.039 (6)
C26	0.226 (14)	0.094 (7)	0.236 (15)	−0.013 (9)	0.131 (12)	−0.010 (9)
C27	0.198 (13)	0.266 (18)	0.224 (15)	0.054 (10)	0.172 (13)	−0.006 (11)
C28	0.182 (12)	0.152 (11)	0.274 (17)	0.074 (10)	0.140 (12)	0.057 (11)
C29	0.179 (12)	0.188 (12)	0.217 (14)	−0.105 (10)	0.108 (11)	−0.072 (10)
C30	0.091 (7)	0.160 (10)	0.269 (16)	−0.038 (7)	0.051 (8)	−0.069 (11)
C31	0.133 (9)	0.232 (15)	0.150 (11)	−0.014 (10)	0.083 (8)	0.057 (11)
C32	0.161 (9)	0.233 (11)	0.163 (9)	−0.005 (8)	0.107 (7)	−0.017 (8)
C35A	0.196 (15)	0.188 (15)	0.194 (15)	0.003 (10)	0.089 (10)	−0.005 (10)
C36A	0.179 (13)	0.182 (14)	0.194 (14)	−0.018 (9)	0.096 (10)	0.015 (9)
C37A	0.159 (11)	0.158 (11)	0.103 (10)	0.028 (9)	0.056 (8)	−0.012 (8)
C38A	0.154 (12)	0.189 (13)	0.152 (12)	0.014 (9)	0.087 (9)	0.005 (9)
C35B	0.148 (12)	0.163 (12)	0.148 (12)	−0.005 (9)	0.080 (9)	0.019 (9)
C36B	0.168 (12)	0.166 (13)	0.168 (13)	−0.009 (9)	0.063 (9)	−0.003 (9)
C37B	0.225 (17)	0.214 (17)	0.209 (17)	0.019 (10)	0.124 (12)	−0.008 (10)
C38B	0.157 (11)	0.142 (11)	0.130 (11)	0.026 (9)	0.077 (8)	−0.002 (8)

### Geometric parameters (Å, °)

**Table:**

Ho1—O1	2.241 (3)	C13—C16	1.363 (10)
Ho1—O3	2.251 (4)	C13—C14	1.376 (10)
Ho1—O7	2.258 (4)	C14—C15	1.394 (11)
Ho1—O5	2.282 (3)	C14—H14	0.9300
Ho1—O4	2.322 (4)	C15—C22	1.360 (15)
Ho1—O6	2.325 (4)	C15—H15	0.9300
Ho1—O2	2.330 (4)	C16—C17	1.393 (11)
Cl1—C2	1.769 (8)	C16—H16	0.9300
Cl2—C2	1.767 (8)	C17—C22	1.333 (15)
Cl3—C2	1.734 (9)	C17—H17	0.9300
Cl4A—C4	1.7493 (11)	C18—C19	1.379 (11)
Cl5A—C4	1.7513 (11)	C18—C20	1.384 (11)
Cl6A—C4	1.7506 (11)	C19—C31	1.433 (13)
Cl4B—C4	1.7508 (11)	C19—H19	0.9300
Cl5B—C4	1.7505 (11)	C20—C21	1.410 (13)
Cl6B—C4	1.7489 (11)	C20—H20	0.9300
Cl7—C6	1.761 (8)	C21—C32	1.326 (18)
Cl8—C6	1.760 (8)	C21—H21	0.9300
Cl9—C6	1.765 (8)	C22—H22	0.9300
P1—O1	1.493 (4)	C23—H23A	0.9600
P1—N1	1.610 (6)	C23—H23B	0.9600
P1—N3	1.613 (6)	C23—H23C	0.9600
P1—N2	1.627 (6)	C24—H24A	0.9600
P2—O3	1.490 (4)	C24—H24B	0.9600
P2—N4	1.615 (6)	C24—H24C	0.9600
P2—N6	1.627 (7)	C25—H25A	0.9600
P2—N5	1.629 (7)	C25—H25B	0.9600
P3—O5	1.484 (4)	C25—H25C	0.9600
P3—N7	1.616 (6)	C26—H26A	0.9600
P3—N9	1.619 (7)	C26—H26B	0.9600
P3—N8	1.629 (8)	C26—H26C	0.9600
P4—O7	1.478 (4)	C27—H27A	0.9600
P4—C18	1.782 (7)	C27—H27B	0.9600
P4—C7	1.784 (6)	C27—H27C	0.9600
P4—C13	1.788 (6)	C28—H28A	0.9600
O2—C1	1.236 (7)	C28—H28B	0.9600
O4—C3	1.242 (7)	C28—H28C	0.9600
O6—C5	1.227 (6)	C29—H29A	0.9600
N1—C1	1.294 (8)	C29—H29B	0.9600
N2—C30	1.438 (11)	C29—H29C	0.9600
N2—C23	1.459 (10)	C30—H30A	0.9600
N3—C25	1.390 (10)	C30—H30B	0.9600
N3—C24	1.472 (11)	C30—H30C	0.9600
N4—C3	1.272 (8)	C31—C32	1.334 (18)
N5—C29	1.390 (12)	C31—H31	0.9300
N5—C26	1.462 (13)	C32—H32	0.9300
N6—C28	1.415 (12)	C35A—H35A	0.9600
N6—C27	1.456 (13)	C35A—H35B	0.9600
N7—C5	1.266 (8)	C35A—H35C	0.9600
N8—C35B	1.3998 (11)	C36A—H36A	0.9600
N8—C35A	1.3999 (11)	C36A—H36B	0.9600
N8—C36B	1.4003 (11)	C36A—H36C	0.9600
N8—C36A	1.4006 (11)	C37A—H37A	0.9600
N9—C38A	1.37 (2)	C37A—H37B	0.9600
N9—C38B	1.3999 (11)	C37A—H37C	0.9600
N9—C37B	1.4004 (11)	C38A—H38A	0.9600
N9—C37A	1.548 (19)	C38A—H38B	0.9600
C1—C2	1.567 (9)	C38A—H38C	0.9600
C3—C4	1.562 (8)	C35B—H35D	0.9600
C5—C6	1.559 (9)	C35B—H35E	0.9600
C7—C8	1.369 (8)	C35B—H35F	0.9600
C7—C12	1.384 (9)	C36B—H36D	0.9600
C8—C9	1.387 (10)	C36B—H36E	0.9600
C8—H8	0.9300	C36B—H36F	0.9600
C9—C10	1.359 (11)	C37B—H37D	0.9600
C9—H9	0.9300	C37B—H37E	0.9600
C10—C11	1.372 (11)	C37B—H37F	0.9600
C10—H10	0.9300	C38B—H38D	0.9600
C11—C12	1.366 (10)	C38B—H38E	0.9600
C11—H11	0.9300	C38B—H38F	0.9600
C12—H12	0.9300		
			
O1—Ho1—O3	163.98 (16)	C8—C7—C12	118.5 (6)
O1—Ho1—O7	78.69 (14)	C8—C7—P4	122.5 (5)
O3—Ho1—O7	116.96 (16)	C12—C7—P4	118.8 (5)
O1—Ho1—O5	105.03 (14)	C7—C8—C9	120.5 (7)
O3—Ho1—O5	82.91 (15)	C7—C8—H8	119.7
O7—Ho1—O5	77.55 (13)	C9—C8—H8	119.7
O1—Ho1—O4	106.45 (15)	C10—C9—C8	120.7 (8)
O3—Ho1—O4	76.30 (15)	C10—C9—H9	119.6
O7—Ho1—O4	75.03 (15)	C8—C9—H9	119.6
O5—Ho1—O4	132.65 (15)	C9—C10—C11	118.7 (8)
O1—Ho1—O6	83.05 (16)	C9—C10—H10	120.7
O3—Ho1—O6	85.63 (17)	C11—C10—H10	120.7
O7—Ho1—O6	141.97 (15)	C12—C11—C10	121.2 (8)
O5—Ho1—O6	75.41 (13)	C12—C11—H11	119.4
O4—Ho1—O6	142.64 (16)	C10—C11—H11	119.4
O1—Ho1—O2	76.26 (13)	C11—C12—C7	120.3 (7)
O3—Ho1—O2	89.75 (15)	C11—C12—H12	119.8
O7—Ho1—O2	131.71 (14)	C7—C12—H12	119.8
O5—Ho1—O2	148.98 (14)	C16—C13—C14	119.4 (7)
O4—Ho1—O2	73.51 (15)	C16—C13—P4	118.5 (5)
O6—Ho1—O2	74.00 (14)	C14—C13—P4	122.1 (6)
O1—P1—N1	116.2 (2)	C13—C14—C15	119.3 (9)
O1—P1—N3	113.1 (3)	C13—C14—H14	120.4
N1—P1—N3	105.7 (3)	C15—C14—H14	120.4
O1—P1—N2	107.2 (3)	C22—C15—C14	120.2 (10)
N1—P1—N2	110.2 (3)	C22—C15—H15	119.9
N3—P1—N2	103.7 (3)	C14—C15—H15	119.9
O3—P2—N4	115.7 (3)	C13—C16—C17	120.3 (9)
O3—P2—N6	107.8 (4)	C13—C16—H16	119.9
N4—P2—N6	109.8 (4)	C17—C16—H16	119.9
O3—P2—N5	114.0 (3)	C22—C17—C16	120.3 (10)
N4—P2—N5	104.0 (4)	C22—C17—H17	119.9
N6—P2—N5	104.9 (4)	C16—C17—H17	119.9
O5—P3—N7	116.6 (2)	C19—C18—C20	119.8 (8)
O5—P3—N9	109.4 (3)	C19—C18—P4	116.9 (6)
N7—P3—N9	107.2 (4)	C20—C18—P4	123.3 (7)
O5—P3—N8	111.4 (3)	C18—C19—C31	119.2 (11)
N7—P3—N8	106.7 (4)	C18—C19—H19	120.4
N9—P3—N8	104.8 (5)	C31—C19—H19	120.4
O7—P4—C18	111.2 (3)	C18—C20—C21	120.3 (11)
O7—P4—C7	110.8 (3)	C18—C20—H20	119.8
C18—P4—C7	108.1 (3)	C21—C20—H20	119.8
O7—P4—C13	110.3 (3)	C32—C21—C20	117.2 (13)
C18—P4—C13	107.5 (3)	C32—C21—H21	121.4
C7—P4—C13	108.9 (3)	C20—C21—H21	121.4
P1—O1—Ho1	135.6 (2)	C17—C22—C15	120.5 (9)
C1—O2—Ho1	135.8 (4)	C17—C22—H22	119.8
P2—O3—Ho1	134.2 (2)	C15—C22—H22	119.8
C3—O4—Ho1	130.8 (4)	N2—C23—H23A	109.5
P3—O5—Ho1	134.6 (2)	N2—C23—H23B	109.5
C5—O6—Ho1	136.7 (4)	H23A—C23—H23B	109.5
P4—O7—Ho1	168.1 (3)	N2—C23—H23C	109.5
C1—N1—P1	123.6 (4)	H23A—C23—H23C	109.5
C30—N2—C23	113.8 (8)	H23B—C23—H23C	109.5
C30—N2—P1	118.2 (7)	N3—C24—H24A	109.5
C23—N2—P1	114.8 (5)	N3—C24—H24B	109.5
C25—N3—C24	111.6 (8)	H24A—C24—H24B	109.5
C25—N3—P1	124.3 (6)	N3—C24—H24C	109.5
C24—N3—P1	116.7 (6)	H24A—C24—H24C	109.5
C3—N4—P2	122.6 (5)	H24B—C24—H24C	109.5
C29—N5—C26	113.0 (9)	N3—C25—H25A	109.5
C29—N5—P2	121.8 (8)	N3—C25—H25B	109.5
C26—N5—P2	118.5 (6)	H25A—C25—H25B	109.5
C28—N6—C27	115.7 (9)	N3—C25—H25C	109.5
C28—N6—P2	123.2 (7)	H25A—C25—H25C	109.5
C27—N6—P2	118.3 (8)	H25B—C25—H25C	109.5
C5—N7—P3	123.4 (4)	N5—C26—H26A	109.5
C35B—N8—C35A	47.7 (14)	N5—C26—H26B	109.5
C35B—N8—C36B	121.5 (16)	H26A—C26—H26B	109.5
C35A—N8—C36B	90.3 (17)	N5—C26—H26C	109.5
C35B—N8—C36A	81.2 (17)	H26A—C26—H26C	109.5
C35A—N8—C36A	111.7 (19)	H26B—C26—H26C	109.5
C36B—N8—C36A	81.0 (15)	N6—C27—H27A	109.5
C35B—N8—P3	124.8 (11)	N6—C27—H27B	109.5
C35A—N8—P3	125.7 (15)	H27A—C27—H27B	109.5
C36B—N8—P3	112.4 (13)	N6—C27—H27C	109.5
C36A—N8—P3	119.9 (13)	H27A—C27—H27C	109.5
C38A—N9—C38B	56.1 (13)	H27B—C27—H27C	109.5
C38A—N9—C37B	52.7 (16)	N6—C28—H28A	109.5
C38B—N9—C37B	101.1 (18)	N6—C28—H28B	109.5
C38A—N9—C37A	119.8 (15)	H28A—C28—H28B	109.5
C38B—N9—C37A	94.6 (12)	N6—C28—H28C	109.5
C37B—N9—C37A	93.1 (18)	H28A—C28—H28C	109.5
C38A—N9—P3	124.0 (13)	H28B—C28—H28C	109.5
C38B—N9—P3	131.5 (11)	N5—C29—H29A	109.5
C37B—N9—P3	114.2 (15)	N5—C29—H29B	109.5
C37A—N9—P3	114.7 (10)	H29A—C29—H29B	109.5
O2—C1—N1	130.7 (6)	N5—C29—H29C	109.5
O2—C1—C2	115.0 (6)	H29A—C29—H29C	109.5
N1—C1—C2	114.3 (6)	H29B—C29—H29C	109.5
C1—C2—Cl3	111.9 (5)	N2—C30—H30A	109.5
C1—C2—Cl2	110.1 (5)	N2—C30—H30B	109.5
Cl3—C2—Cl2	107.5 (4)	H30A—C30—H30B	109.5
C1—C2—Cl1	108.8 (5)	N2—C30—H30C	109.5
Cl3—C2—Cl1	110.2 (5)	H30A—C30—H30C	109.5
Cl2—C2—Cl1	108.2 (4)	H30B—C30—H30C	109.5
O4—C3—N4	131.8 (6)	C32—C31—C19	117.1 (13)
O4—C3—C4	113.2 (5)	C32—C31—H31	121.4
N4—C3—C4	114.9 (5)	C19—C31—H31	121.4
C3—C4—Cl6B	116.5 (4)	C21—C32—C31	126.4 (15)
C3—C4—Cl4A	112.1 (5)	C21—C32—H32	116.8
Cl6B—C4—Cl4A	123.3 (5)	C31—C32—H32	116.8
C3—C4—Cl5B	109.3 (5)	N8—C35A—H35A	109.5
Cl6B—C4—Cl5B	110.3 (5)	N8—C35A—H35B	109.5
C3—C4—Cl6A	104.3 (5)	N8—C35A—H35C	109.5
Cl6B—C4—Cl6A	84.2 (5)	N8—C36A—H36A	109.5
Cl4A—C4—Cl6A	110.4 (5)	N8—C36A—H36B	109.5
Cl5B—C4—Cl6A	130.9 (6)	N8—C36A—H36C	109.5
C3—C4—Cl4B	108.1 (6)	N9—C37A—H37A	109.5
Cl6B—C4—Cl4B	103.0 (6)	N9—C37A—H37B	109.5
Cl4A—C4—Cl4B	87.5 (5)	N9—C37A—H37C	109.5
Cl5B—C4—Cl4B	109.4 (5)	N9—C38A—H38A	109.5
C3—C4—Cl5A	109.1 (5)	N9—C38A—H38B	109.5
Cl4A—C4—Cl5A	108.8 (5)	N9—C38A—H38C	109.5
Cl5B—C4—Cl5A	89.6 (4)	N8—C35B—H35D	109.5
Cl6A—C4—Cl5A	112.1 (5)	N8—C35B—H35E	109.5
Cl4B—C4—Cl5A	129.1 (6)	N8—C35B—H35F	109.5
O6—C5—N7	131.8 (6)	N8—C36B—H36D	109.5
O6—C5—C6	114.1 (5)	N8—C36B—H36E	109.5
N7—C5—C6	114.1 (5)	N8—C36B—H36F	109.5
C5—C6—Cl8	112.2 (4)	N9—C37B—H37D	109.5
C5—C6—Cl7	109.8 (5)	N9—C37B—H37E	109.5
Cl8—C6—Cl7	107.5 (4)	N9—C37B—H37F	109.5
C5—C6—Cl9	109.6 (5)	N9—C38B—H38D	109.5
Cl8—C6—Cl9	108.6 (4)	N9—C38B—H38E	109.5
Cl7—C6—Cl9	109.0 (4)	N9—C38B—H38F	109.5
			
N1—P1—O1—Ho1	−2.0 (5)	N9—P3—N8—C36B	40.4 (14)
N3—P1—O1—Ho1	120.6 (4)	O5—P3—N8—C36A	−108.9 (15)
N2—P1—O1—Ho1	−125.7 (4)	N7—P3—N8—C36A	19.3 (16)
O3—Ho1—O1—P1	−20.6 (8)	N9—P3—N8—C36A	132.8 (15)
O7—Ho1—O1—P1	147.6 (4)	O5—P3—N9—C38A	29.1 (19)
O5—Ho1—O1—P1	−138.7 (3)	N7—P3—N9—C38A	−98.2 (18)
O4—Ho1—O1—P1	77.1 (4)	N8—P3—N9—C38A	148.7 (18)
O6—Ho1—O1—P1	−65.9 (4)	O5—P3—N9—C38B	−43.4 (17)
O2—Ho1—O1—P1	9.2 (3)	N7—P3—N9—C38B	−170.7 (16)
O1—Ho1—O2—C1	−12.7 (5)	N8—P3—N9—C38B	76.2 (17)
O3—Ho1—O2—C1	159.5 (5)	O5—P3—N9—C37B	89.2 (18)
O7—Ho1—O2—C1	−73.4 (6)	N7—P3—N9—C37B	−38.1 (18)
O5—Ho1—O2—C1	83.7 (6)	N8—P3—N9—C37B	−151.2 (18)
O4—Ho1—O2—C1	−124.8 (6)	O5—P3—N9—C37A	−165.0 (10)
O6—Ho1—O2—C1	73.9 (5)	N7—P3—N9—C37A	67.7 (11)
N4—P2—O3—Ho1	−5.4 (6)	N8—P3—N9—C37A	−45.4 (12)
N6—P2—O3—Ho1	−128.8 (4)	Ho1—O2—C1—N1	6.4 (10)
N5—P2—O3—Ho1	115.3 (5)	Ho1—O2—C1—C2	−174.0 (4)
O1—Ho1—O3—P2	90.7 (7)	P1—N1—C1—O2	8.8 (10)
O7—Ho1—O3—P2	−76.3 (4)	P1—N1—C1—C2	−170.7 (5)
O5—Ho1—O3—P2	−148.4 (4)	O2—C1—C2—Cl3	4.9 (8)
O4—Ho1—O3—P2	−11.2 (4)	N1—C1—C2—Cl3	−175.4 (5)
O6—Ho1—O3—P2	135.8 (4)	O2—C1—C2—Cl2	124.5 (5)
O2—Ho1—O3—P2	61.8 (4)	N1—C1—C2—Cl2	−55.9 (7)
O1—Ho1—O4—C3	−132.9 (6)	O2—C1—C2—Cl1	−117.0 (5)
O3—Ho1—O4—C3	30.8 (6)	N1—C1—C2—Cl1	62.6 (7)
O7—Ho1—O4—C3	154.0 (6)	Ho1—O4—C3—N4	−32.6 (12)
O5—Ho1—O4—C3	97.3 (6)	Ho1—O4—C3—C4	151.1 (4)
O6—Ho1—O4—C3	−32.6 (7)	P2—N4—C3—O4	1.1 (12)
O2—Ho1—O4—C3	−63.1 (6)	P2—N4—C3—C4	177.3 (4)
N7—P3—O5—Ho1	−10.0 (5)	O4—C3—C4—Cl6B	177.1 (5)
N9—P3—O5—Ho1	−131.8 (5)	N4—C3—C4—Cl6B	0.1 (8)
N8—P3—O5—Ho1	112.8 (5)	O4—C3—C4—Cl4A	−33.0 (7)
O1—Ho1—O5—P3	91.2 (4)	N4—C3—C4—Cl4A	150.1 (6)
O3—Ho1—O5—P3	−74.6 (4)	O4—C3—C4—Cl5B	−57.1 (7)
O7—Ho1—O5—P3	165.7 (4)	N4—C3—C4—Cl5B	126.0 (6)
O4—Ho1—O5—P3	−138.6 (3)	O4—C3—C4—Cl6A	86.5 (7)
O6—Ho1—O5—P3	12.7 (3)	N4—C3—C4—Cl6A	−90.4 (7)
O2—Ho1—O5—P3	2.9 (5)	O4—C3—C4—Cl4B	61.9 (7)
O1—Ho1—O6—C5	−119.6 (7)	N4—C3—C4—Cl4B	−115.1 (7)
O3—Ho1—O6—C5	71.8 (7)	O4—C3—C4—Cl5A	−153.5 (5)
O7—Ho1—O6—C5	−58.0 (8)	N4—C3—C4—Cl5A	29.5 (7)
O5—Ho1—O6—C5	−12.0 (7)	Ho1—O6—C5—N7	7.3 (14)
O4—Ho1—O6—C5	132.3 (7)	Ho1—O6—C5—C6	−171.9 (5)
O2—Ho1—O6—C5	162.8 (7)	P3—N7—C5—O6	3.1 (13)
C18—P4—O7—Ho1	−86.1 (13)	P3—N7—C5—C6	−177.8 (5)
C7—P4—O7—Ho1	34.1 (14)	O6—C5—C6—Cl8	2.5 (9)
C13—P4—O7—Ho1	154.8 (13)	N7—C5—C6—Cl8	−176.9 (6)
O1—Ho1—O7—P4	142.0 (13)	O6—C5—C6—Cl7	121.9 (6)
O3—Ho1—O7—P4	−41.6 (14)	N7—C5—C6—Cl7	−57.4 (8)
O5—Ho1—O7—P4	33.7 (13)	O6—C5—C6—Cl9	−118.3 (6)
O4—Ho1—O7—P4	−107.3 (13)	N7—C5—C6—Cl9	62.4 (8)
O6—Ho1—O7—P4	79.2 (14)	O7—P4—C7—C8	107.8 (6)
O2—Ho1—O7—P4	−158.1 (12)	C18—P4—C7—C8	−130.1 (6)
O1—P1—N1—C1	−10.4 (7)	C13—P4—C7—C8	−13.7 (7)
N3—P1—N1—C1	−136.8 (6)	O7—P4—C7—C12	−66.3 (6)
N2—P1—N1—C1	111.8 (6)	C18—P4—C7—C12	55.7 (6)
O1—P1—N2—C30	−174.1 (8)	C13—P4—C7—C12	172.2 (6)
N1—P1—N2—C30	58.6 (9)	C12—C7—C8—C9	−0.6 (11)
N3—P1—N2—C30	−54.2 (9)	P4—C7—C8—C9	−174.7 (6)
O1—P1—N2—C23	47.2 (7)	C7—C8—C9—C10	0.0 (13)
N1—P1—N2—C23	−80.1 (7)	C8—C9—C10—C11	−0.6 (14)
N3—P1—N2—C23	167.2 (7)	C9—C10—C11—C12	1.8 (14)
O1—P1—N3—C25	−98.2 (8)	C10—C11—C12—C7	−2.4 (14)
N1—P1—N3—C25	30.0 (9)	C8—C7—C12—C11	1.8 (12)
N2—P1—N3—C25	146.0 (8)	P4—C7—C12—C11	176.1 (7)
O1—P1—N3—C24	49.0 (8)	O7—P4—C13—C16	−25.7 (6)
N1—P1—N3—C24	177.2 (7)	C18—P4—C13—C16	−147.0 (6)
N2—P1—N3—C24	−66.8 (8)	C7—P4—C13—C16	96.1 (6)
O3—P2—N4—C3	17.4 (7)	O7—P4—C13—C14	154.8 (6)
N6—P2—N4—C3	139.8 (6)	C18—P4—C13—C14	33.5 (7)
N5—P2—N4—C3	−108.4 (6)	C7—P4—C13—C14	−83.4 (6)
O3—P2—N5—C29	65.3 (9)	C16—C13—C14—C15	0.1 (12)
N4—P2—N5—C29	−167.7 (8)	P4—C13—C14—C15	179.6 (7)
N6—P2—N5—C29	−52.4 (9)	C13—C14—C15—C22	2.0 (15)
O3—P2—N5—C26	−84.0 (8)	C14—C13—C16—C17	−1.2 (12)
N4—P2—N5—C26	43.0 (8)	P4—C13—C16—C17	179.3 (7)
N6—P2—N5—C26	158.3 (8)	C13—C16—C17—C22	0.1 (15)
O3—P2—N6—C28	15.8 (10)	O7—P4—C18—C19	−29.2 (6)
N4—P2—N6—C28	−111.1 (10)	C7—P4—C18—C19	−151.0 (6)
N5—P2—N6—C28	137.6 (10)	C13—P4—C18—C19	91.6 (6)
O3—P2—N6—C27	176.3 (8)	O7—P4—C18—C20	152.3 (6)
N4—P2—N6—C27	49.4 (9)	C7—P4—C18—C20	30.5 (7)
N5—P2—N6—C27	−61.8 (9)	C13—P4—C18—C20	−86.9 (6)
O5—P3—N7—C5	−1.5 (8)	C20—C18—C19—C31	0.4 (12)
N9—P3—N7—C5	121.5 (7)	P4—C18—C19—C31	−178.2 (7)
N8—P3—N7—C5	−126.7 (7)	C19—C18—C20—C21	0.2 (12)
O5—P3—N8—C35B	−8.3 (18)	P4—C18—C20—C21	178.6 (6)
N7—P3—N8—C35B	120.0 (17)	C18—C20—C21—C32	0.0 (16)
N9—P3—N8—C35B	−126.5 (17)	C16—C17—C22—C15	2.0 (18)
O5—P3—N8—C35A	51 (2)	C14—C15—C22—C17	−3.1 (18)
N7—P3—N8—C35A	179.3 (19)	C18—C19—C31—C32	−1.2 (17)
N9—P3—N8—C35A	−67 (2)	C20—C21—C32—C31	−1(2)
O5—P3—N8—C36B	158.7 (13)	C19—C31—C32—C21	2(2)
N7—P3—N8—C36B	−73.0 (14)		
